# Deactivation of catalysts in simultaneous reversible and irreversible parahydrogen NMR signal enhancement, and the role of co-ligands in the stabilization of the reversible method†

**DOI:** 10.1039/d2ra02872g

**Published:** 2022-05-27

**Authors:** Adam Mames, Sylwia Jopa, Mariusz Pietrzak, Tomasz Ratajczyk

**Affiliations:** Institute of Physical Chemistry, Polish Academy of Sciences Kasprzaka 44/52 Warsaw 01-224 Poland tratajczyk@ichf.edu.pl; Faculty of Chemistry, University of Warsaw Pasteura 1 Warsaw 02-093 Poland

## Abstract

Signal Amplification by Reversible Exchange (SABRE) and hydrogeneable Parahydrogen Induced Polarization (hPHIP) can enhance weak NMR signals, and thus increase the range of NMR applications. Here, using an N-heterocyclic carbene Ir-based catalyst, simultaneous SABRE and hPHIP was achieved for the compound with an N-donor site and an acetylene triple bond. It was demonstrated that the interplay between SABRE and hPHIP can be manipulated. Specifically, it was found that the hPHIP effect could be almost completely suppressed, while stable SABRE was observed in subsequent consecutive experiments. The presented results have the potential to increase the numbers of parahydrogen hyperpolarizable molecules.

## Abstract 2

Signal Amplification by Reversible Exchange (SABRE) and hydrogeneable Parahydrogen Induced Polarization (hPHIP) can enhance weak NMR signals, and thus increase the range of NMR applications.

## Graphical abstract

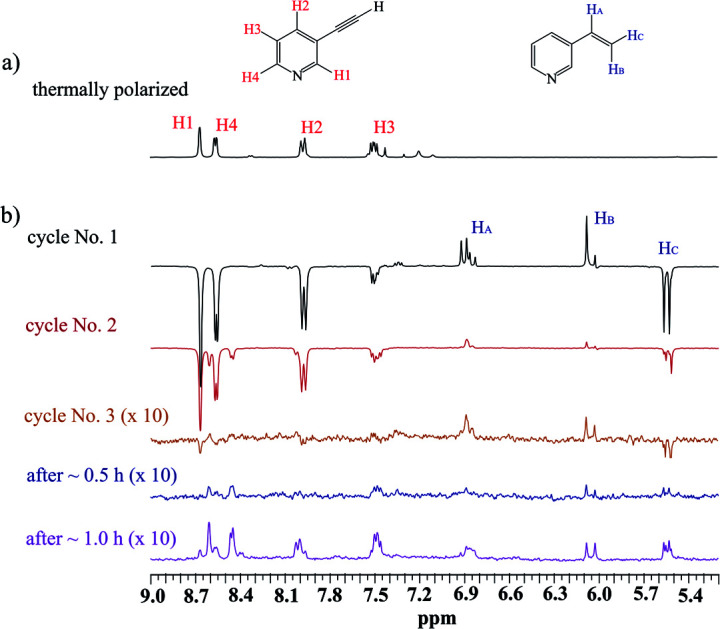

Analytical methods that can deliver comprehensive information about broadly understood molecular and biological systems, and materials which are built from them, are of central importance for progress in various fields of science, medicine, and industry. There is no doubt that NMR is one of the most powerful analytical methods. It would be a challenge to enumerate all of the applications and advantages of NMR. It particularly offers in-depth insight into the molecular structures of both small organic molecules and complex functional biomolecular systems.^1,2^ NMR can be used for the monitoring and investigation of many multiple processes in which molecules participate, such as diffusion and various aspects of molecular dynamics.^3–5^ Magnetic Resonance Imaging (MRI) of living organisms is common in human and veterinary medicine, where MRI serves as a powerful tool for the identification of various dysfunctions, and often MRI can deliver a medical diagnosis of cancer in its early stages.^6,7^ NMR is also being more and more often employed in different branches of industry; for example, for product quality control in the food industry, the petrochemical field, pharmacy and many other fields.^8–11^ After mentioning the countless applications and advantages of NMR, one also has to mention the severe limitations of MR based methods. Precisely, MR suffers from inherently low sensitivity, which results from low nuclear spin polarization.^12,13^ As a consequence, MR methods cannot be employed for the investigation of important systems in which investigated nuclei are at low concentrations. MR methods are also usually expensive due to the high costs of the equipment and their maintenance. Thus, since the early days of NMR, the low sensitivity of this method has been one of the main concerns, and many approaches have been developed to overcome the low sensitivity of NMR.^13–16^ One of the most powerful approaches is hyperpolarization, in which nuclear spin polarization is enhanced *via* polarization transfer from the degree of freedom, where the polarization is high, to the nuclear spins.^12,14^ For example, the *p*-H_2_ hydrogen gas mixture enriched in a parahydrogen isomer in relation to the mixture from thermal equilibrium at room temperature is a reservoir of hyperpolarization.^17^ This hyperpolarization can be relocated to another molecular system. Two variants of Parahydrogen Induced Polarization (PHIP) transfer are possible: irreversible and reversible. The first variant, a hydrogenable Parahydrogen Induced Polarization (hPHIP) involves metal-catalyzed hydrogenation or hydroformylation reactions of unsaturated bonds *via p*-H_2_.^18–20^ According to the reversible variant of PHIP, the NMR signal is amplified by reversible exchange (SABRE).^21,22^ In SABRE, a labile ternary complex between the *p*-H_2_, the pre-catalyst and the to-be-hyperpolarized ligand is created. In this complex, the hyperpolarization is transferred to the ligand *via* the freshly activated metal catalyst. Thus, when the ligand is released from the labile structure, it is hyperpolarized.

hPHIP and SABRE have already been demonstrated to be capable of enhancing the analytical potential of NMR.^23,24^ Both techniques have become very efficient methods for hyperpolarization of various molecular systems with biological relevance.^25–29^ In particular, several PHIP hyperpolarized molecular systems can be; for example, employed as MRI contrast agents.^30–32^ PHIP also has potential in the field of metabolomics and the monitoring of chemical reactions where traces of various analytes can be detected.^33–36^

SABRE and hPHIP are considered to be separate methods. However, the combination of different scientific approaches or analytical techniques can very often culminate in interesting results, or deliver information that cannot be obtained *via* one individual method alone. Simultaneous SABRE and PHIP hyperpolarization may deliver a higher NMR signal enhancement per molecule unit. This could enhance the NMR sensitivity.

Thus, we performed a simultaneous SABRE and hPHIP experiment for the same compound. Coming straight to the point of our research, we wanted to see what would happen when we attempt to hyperpolarize a 3-ethynylpyridine (1) molecule simultaneously *via* SABRE and hPHIP (see Scheme 1). 1 was chosen because it contains two fragments: pyridine, which should facilitate SABRE, and acetylene units, which can be hydrogenated, allowing hPHIP to occur.

**Scheme 1:**
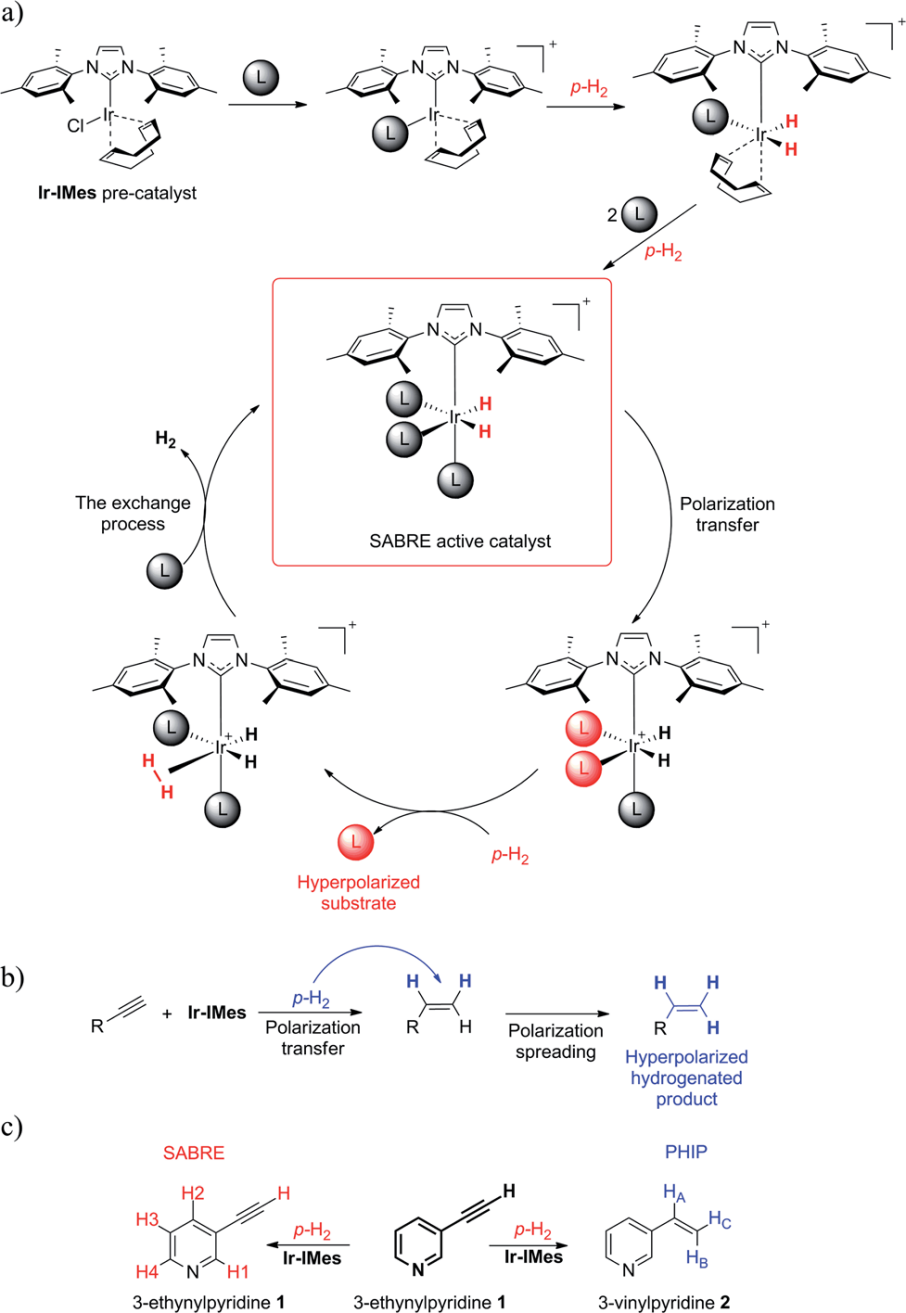
(a) The Ir-IMes pre-catalyst and its activation, which leads to the ternary labile SABRE complex, where reversible exchange reaction produces a hyperpolarized ligand; (b) a scenario of hPHIP on the example of a molecule with an unsaturated triple bond; (c) our exemplary model of 3-ethynylpyridine (1) which can potentially undergo both processes, SABRE and hPHIP.

As a pre-catalyst, we employed [Ir(COD)(IMes)Cl] (where IMes is the N-heterocyclic carbene ligand = 1,3-bis(2,4,6-trimethyl-phenyl)imidazol-2-ylidene and COD = 1,5-cyclooctadiene), which is used in the vast majority of the research concerned with SABRE, but which can also facilitate hydrogenation (for the sake of convenience it is referred to here as Ir-IMes).^37–40^

Having prepared a sample with 1 and Ir-IMes dissolved in MeOD-d_4_, we started hyperpolarization experiments immediately (see ESI†). The *p*-H_2_ was injected, the sample was mixed vigorously at the Earth's magnetic field (see ESI†), the NMR tube was inserted into the magnet, and ^1^H NMR spectra were acquired. For the purpose of this manuscript, we will call this type of NMR spectra *p*-H_2_^1^H NMR spectra. Afterwards, the NMR sample was allowed to relax for 60 seconds. Then, the following ^1^H NMR spectrum, which will be called a relaxed spectrum, was recorded. Finally, the sample was ejected from the spectrometer, and the entire procedure, which will be called a cycle, was repeated.

The analysis of the spectra from the first cycle revealed simultaneous hPHIP and SABRE hyperpolarization. Regarding SABRE, the signals derived from hydrogen atoms of the pyridine unit at 8.66 (H1), 8.55 (H4), 7.95 (H2), 7.46 (H3) ppm were negative, and their enhancement factors (EF) were 5.2, 5.0, 4.1 and 1.1 for H1, H4, H2, and H3, respectively (see ESI† for the calculations of EF). Despite the effort which has been made to do manual experiments in exactly the same way, the discrepancy between the same cycles for several samples which were prepared in the same manner can be significant, as it can reach even 50%. The value of the EF decreases in the order H1, H4, H2, with the lowest value being for H3.

Also, signals in the hydride region were observed at −12.3 and −17.6 ppm (see ESI†). Signals with similar chemical shifts have been observed several times by different research groups.^41,42^ The appearance of such signals is usually connected with the activation process of the Ir-IMes pre-catalyst. Apart from SABRE, the signals concerned with hPHIP were observed at 6.82 (H_A_), 5.97 (H_B_) and 5.45 (H_C_) ppm. The position of these signals are the same as for three vinyl protons from 3-vinylpyridine (2) (see Scheme 1). Thus, 1 is hydrogenated into 2.

The ^1^H NMR pattern has typical hPHIP features.^17^ Thus, hydrogenation occurred when the sample was shaken at the Earth's magnetic field, *i.e.*, at the same time when SABRE took place. The hPHIP enhancement factors for the hyperpolarized 2 were 9.7, 21.1, 27.8 for H_A_, H_B_, H_C_, vinyl protons, respectively.

The scenario of the above-described hyperpolarization experiments was repeated several times. One could expect that the activation of the SABRE pre-catalyst would continue; if so, the next consecutive experiments should have delivered more significant SABRE, while hPHIP hyperpolarization would be expected to be on a similar level (or lower because the substrate is consumed during the hydrogenation). The analysis of several consecutive cycles revealed, however, that both SABRE and hPHIP decreased step-by-step in the consecutive cycles unit. Finally, we were not able to observe either SABRE or hPHIP, despite the fact that the substrate for hydrogenation was still present in the sample. Afterwards, the sample was kept under the *p*-H_2_ atmosphere without mixing for 30 minutes, and also for 1 hour (Fig. 1). Surprisingly, the hyperpolarization effect was not visible after this time. During the hydrogenation processes which take place during the shaking procedure, the H_2_ gas was consumed and it could influence the SABRE effect. However, the amount of hydrogen which was consumed for the hydrogenation is very low in comparison to the amount of the substrate (see ESI†).

**Fig. 1:**
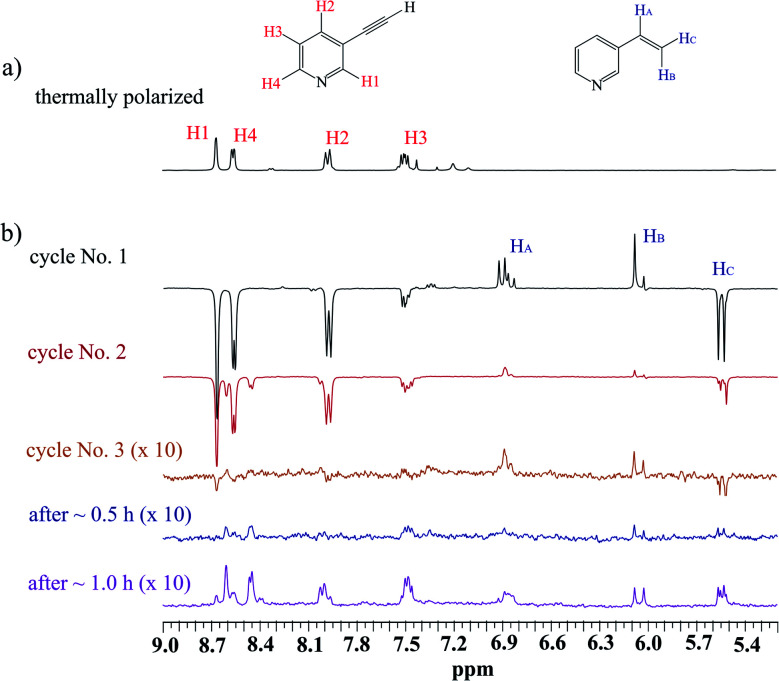
The ^1^H NMR spectra of the mixture of 1 with the 10 mol% Ir-IMes pre-catalyst in MeOD-d_4_ at 298 K; (a) before *p*-H_2_ administration (b) several consecutive hyperpolarization experiments.

It seems that some deactivation processes took place in the sample, and as a result, no Ir SABRE and also no hPHIP active species were present in the sample after some time.

In order to gain insight into this unexpected situation, we have taken a closer look at the first step of the process which is concerned with the activation of the catalyst, *i.e.*, the interaction between Ir-IMes pre-catalyst and the ligand before the administration of hydrogen gas. The nature of this interaction is similar to the previously reported cases, *i.e.*, the Ir-IMes pre-catalyst coordinates the ligand. It was observed that not all Ir-IMes were involved in the formation of the complex, even though the ligand is used quite excessively. This situation was also observed for some ligands (for example, 4-methylpyridyne or pyridine labeled oligopeptides) when they were used for SABRE, and they were efficiently hyperpolarized without the deactivation processes that we observed in the case of 1.^35,43^ Thus, at this step, no adverse effect occurs which could result in SABRE deactivation.

According to the literature data, several examples of the deactivation of hyperpolarization were observed in the case of SABRE experiments with specific ligands.^44–46^ For example, it was observed that for 5-(tributylstannyl)pyrimidine and 5-(trimethylsilyl)pyrimidine, a typical ternary labile SABRE complex was formed, and hyperpolarization was observed.^44^ However, the ternary labile SABRE complexes can conglomerate into tri-metal-center structures which are SABRE inactive (see ESI Fig. S9†). Also, in the case of the SABRE hyperpolarization of oxalate, the formation of the SABRE inactive dimer with two iridium centers was assumed to be responsible for the inefficient hyperpolarization.^46^ Notably, the polymerization was also observed in the case of vinyl sulfoxides, which form a reversible one Ir atom center complex with Ir-IMes and *p*-H_2_ in which the hydrogenation of vinyl bonds occurs (see ESI Fig. S10†).^45^ However, due to the dimerization of this complex, the dimer exhibiting irreversible nature and no hydrogenable properties was formed, and thus deactivation of the PHIP active catalyst was observed.

Therefore, we hypothesize that in the case of 1, the SABRE and hPHIP deactivation processes can also be caused by polymerization or decomposition of the initial active catalyst.

In the case of 1, only one nitrogen atom is accessible for coordination with the iridium atom. However, there is also an unsaturated triple bond which can hypothetically bind to another Ir coordination center. Thus, in principle, the structure of 1 can facilitate a polymerization process, which can be responsible for the damping of SABRE activity. Or, more generally, the initial SABRE-hPHIP active species rearrange into inactive ones.

Thus, there is the interesting question of what will happen when the Ir coordination sphere in which unwanted deactivation occurs is modified by additional ligands (*i.e.*, co-ligands). This is important, because the co-ligand can limit the number of 1 molecules within the coordination sphere; as one or two coordination positions will be occupied by the co-ligand. Thus, the modification of the coordination sphere can potentially minimize the adverse effect of the SABRE deactivation process.

To determine whether the modification of the Ir coordination sphere within the SABRE complex can modify the SABRE activity, we did a simple experiment with a co-ligand, namely dimethyl sulfoxide (DMSO). DMSO does not have any NMR signals in the vinyl and aromatic region. Therefore, it can be considered as SABRE and hPIHP “silent”, but it can participate in the hPHIP and SABRE mechanisms.^46,48^ The chemistry of the DMSO activation of the Ir-IMes pre-catalyst has been already described.^46–48^ Specifically, the activation with DMSO leads to the [IrCl(IMes)(H)_2_ (DMSO)_2_] complex (see Scheme 2). This complex can incorporate another ligand (L) – for example, 3-ethynylpyridine or 2,5-lutidine.

**Scheme 2:**
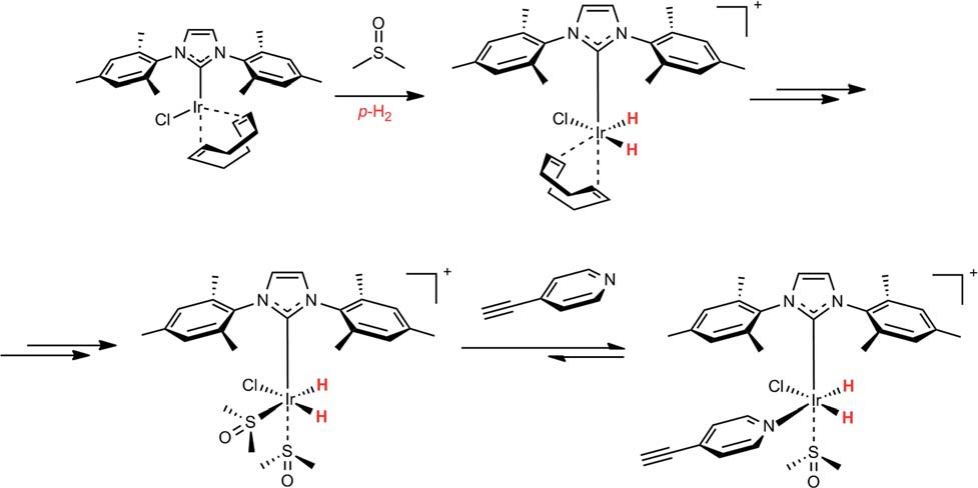
A basic scenario of the activation of the Ir-IMes pre-catalyst with DMSO, and the consecutive reaction of the activated Ir complex with 1.

As a result, [IrCl(IMes)(H)_2_(L)(DMSO)] is created, in which only one molecule of that ligand will be in the coordination sphere of iridium atom. Importantly, in this complex, the SABRE of the ligand is possible.

Having had the DMSO activated sample, 1 was loaded to this sample, and hyperpolarization experiments were conducted. Fig. 2 displays spectra concerned with several consecutive cycles and after 0.5 and 1.0 hours after the first cycle. While signals concerned with hPHIP of 1 were very tiny (see ESI†), the aromatic protons of 1 displayed the SABRE pattern with the enhancement 29.7, 28.0, 21.8 and 5.39 for protons H1, H4, H2 and H3, respectively. Thus, the hPHIP effect was switched off and SABRE was switched on *via* the addition of DMSO during the activation of the pre-catalyst. Therefore, the SABRE hyperpolarization which involves the activation of the co-ligand is more efficient than the direct SABRE hyperpolarization. Importantly, several consecutive cycles revealed that the SABRE hyperpolarization was observed for a longer time. The SABRE activity was checked after 30 minutes and again after 1 hour, and the polarization effect was still observed. However, the SABRE activity decreased over time.

**Fig. 2:**
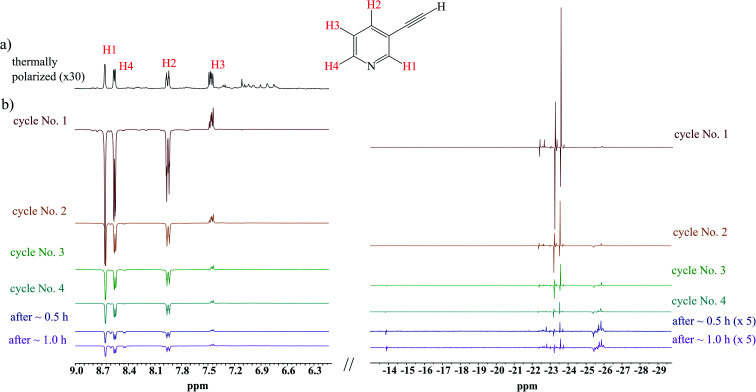
(a) The ^1^H NMR spectra of the mixture of 1 with 10 mol% DMSO as a co-ligand and the pre-activated 10 mol% Ir-IMes pre-catalyst in MeOD-d_4_ at 298 K; (b) several consecutive hyperpolarization experiments.

The analysis of hydride region revealed two hydride signals at −23.5 and −23.7 ppm. These signals can be assigned to the complex [Ir(IMes)(H)_2_ (1)(DMSO)Cl], in which a polarization was transferred to 1 (see Scheme 2). It is worth mentioning that an analogous complex with 2,5-lutidine was observed by Ranier.^47^ The intensities of the hydride signals were decreasing over time. Thus, the concentration of the effective SABRE complex was also decreasing, which was reflected by the steadily decreasing signal enhancement of the ^1^H signals in the pyridine unit in the consecutive cycles. Moreover, the decrease of hyperpolarization efficiency and hydride resonance intensities had a similar character, as is shown in the decay curves (see ESI†). Apart from the signals at −23.5 and −23.7 ppm, several additional hydride resonances were also observed. In particular, the signals at −14.1 and −22.9 ppm could be assigned to the complex [Ir(IMes)(H)_2_ (DMSO)_2_Cl].^47,48^

In conclusion, the Ir-IMes catalyst can simultaneously deliver SABRE and hPHIP for a molecule that contains N-donor units which are suitable for reversible exchange, and unsaturated bonds which make hydrogenation possible. Such a structure can potentially facilitate deactivation *via* the polymerization of Ir SABRE and hPHIP active species, as the multimeric species usually do not induce reversible exchange and hydrogenation. The mechanism of simultaneous SABRE and hPHIP, and thus the interplay between SABRE and hPHIP, can be manipulated *via* the initial activation of the catalyst. This activation procedure almost entirely suppresses hPHIP efficiency, whereas SABRE can be carried out multiple times. These results also demonstrate that additional molecules which do not undergo any reaction within the catalytic cycle can modify the properties of metal-based catalysts towards new functions. Finally, the interplay between SABRE and hPHIP in the context of molecular structures and pre-catalyst activation poses an issue for further investigation. This can be of importance for further SABRE and hPHIP applications in hyperpolarization of more biorelevant molecules containing motifs similar to that in 1. The presented results can also be helpful during the SABRE hyperpolarization of systems in which unsaturated bonds cause a suppression of the SABRE effect. Our work in this area is steadily progressing.

## Conflicts of interest

Authors declare no conflict of interests.

## Supplementary Material

RA-012-D2RA02872G-s001
