# Supplementary material for: Deactivation of catalysts in simultaneous reversible and irreversible parahydrogen NMR signal enhancement, and the role of co-ligands in the stabilization of the reversible method
Source: RSC Adv. 2022 May 27;12(25):15986–91. doi: 10.1039/d2ra02872g (PMC9136854; doi:10.1039/d2ra02872g)
Supplement: RA-012-D2RA02872G-s001 [file RA-012-D2RA02872G-s001.pdf]

Electronic Supplementary Material (ESI) for RSC Advances.
This journal is © The Royal Society of Chemistry 2022

Deactivation of catalysts in simultaneous reversible and irreversible parahydrogen NMR signal enhancement, and the role of co-ligands in the stabilization of the reversible method.

Adam Mames [a], Sylwia Jopa [b], Mariusz Pietrzak [a] and Tomasz Ratajczyk [a*]

a. Institute of Physical Chemistry, Polish Academy of Sciences, Kasprzaka 44/52, 01-224 Warsaw, Poland

b. Faculty of Chemistry, University of Warsaw, Pasteura 1, 02-093 Warsaw, Poland

Table of Contents

General information: Materials and methods. 2

Commercially available chemicals. 2

Synthesis of the [IrCl(COD)(IMes)] pre-catalyst. 3

NMR spectrometer. 3

The investigation of interaction between Ir-IMes and 3-ethynylpyridine. 4

Parahydrogen enrichment. 6

Hyperpolarization. 6

Sample preparation for NMR hyperpolarization experiments without DMSO as co-ligand .6

Sample preparation for NMR hyperpolarization experiments with DMSO as a co-ligand 6

The definition of cycle. 6

Hyperpolarization without DMSO as a co-ligand. 9

The deactivation of SABRE ternary complexes via dimerization and polymerization. 11

Hyperpolarization with DMSO as a co-ligand. 12

The decay of SABRE activity. 15

References. 17

General information: Materials and methods.

Commercially available chemicals.

Commercially available chemicals were purchased from Sigma-Aldrich and used without any further purification. In particular, 3-ethynylpyridine (CAS Number: 2510-23-8, Sigma-Aldrich product code 520446-1G) was purchased from Sigma-Aldrich and used without any purifications (see Fig. S1 for the ^{1}H NMR of purchased 3-ethynylpyridine). Deuterated solvents were purchased from EUROISOTOP, and they were also used as obtained.

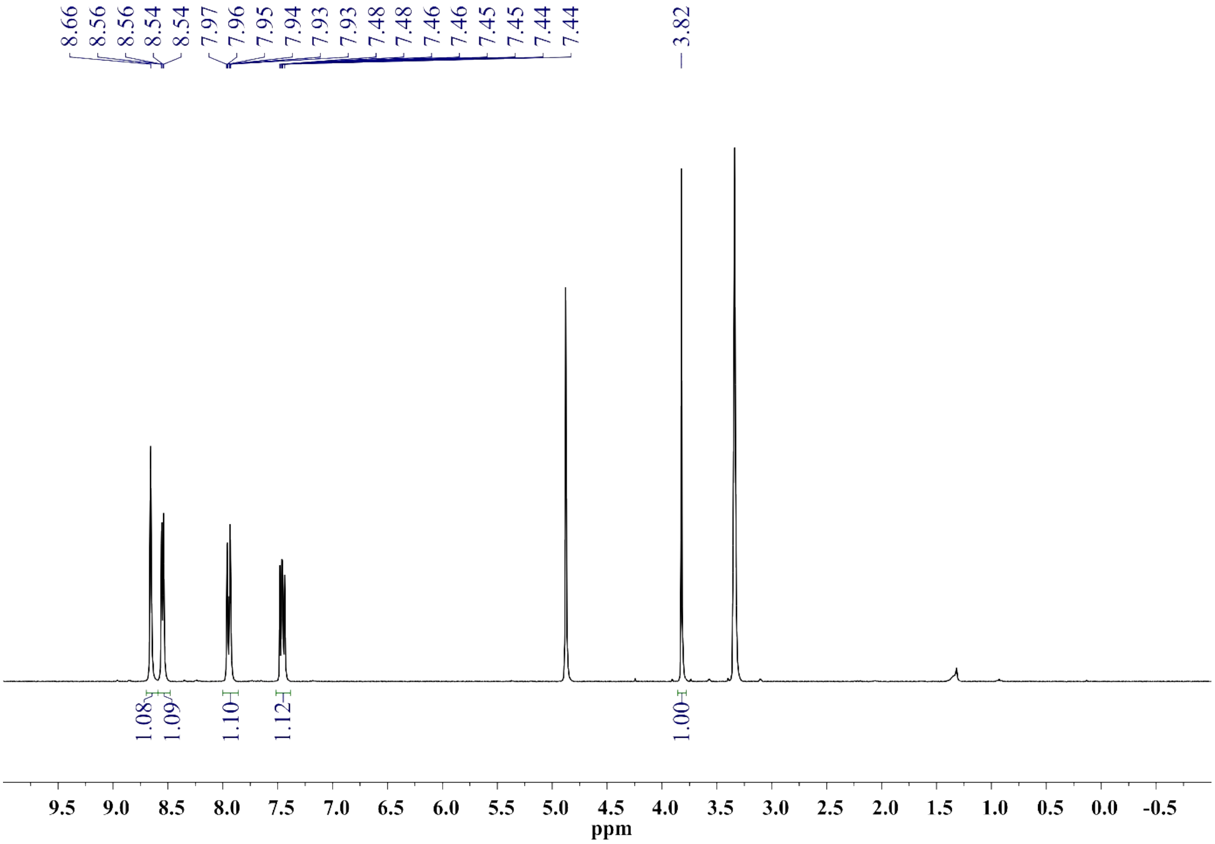

Fig. S1 ^{1}H NMR spectrum of 3-ethynylpyridine purchased from Sigma-Aldrich in MeOD-d_4.

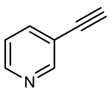

3-Ethynylpyridine (**1**) ^{1}H NMR (300 MHz, MeOD-d_4) δ (ppm) 8.66 (d, *J* = 1.3 Hz, 1H), 8.55 (dd, *J* = 1.5, 5.0 Hz, 1H), 8.00 – 7.90 (m, 1H), 7.46 (ddd, *J* = 7.9, 5.0, 0.7 Hz, 1H), 3.83 (s, 1H).

Synthesis of the [IrCl(COD)(IMes)] pre-catalyst.

The [IrCl(COD)(IMes)] (**Ir-IMes**) pre-catalyst is the most efficient catalyst that is used in SABRE experiments. However, it is commercially not available. Thus, this catalyst was prepared and purified in accordance with the procedure described by Kownacki, et al.[1] The structure of the obtained [IrCl(COD)(IMes)] pre-catalyst was confirmed by NMR spectroscopy (see Fig. S2 for the ^{1}H NMR spectra of the **Ir-IMes** pre-catalyst prepared by us) i.e., the ^{1}H NMR spectra was the same as the spectra available in the literature [1,2].

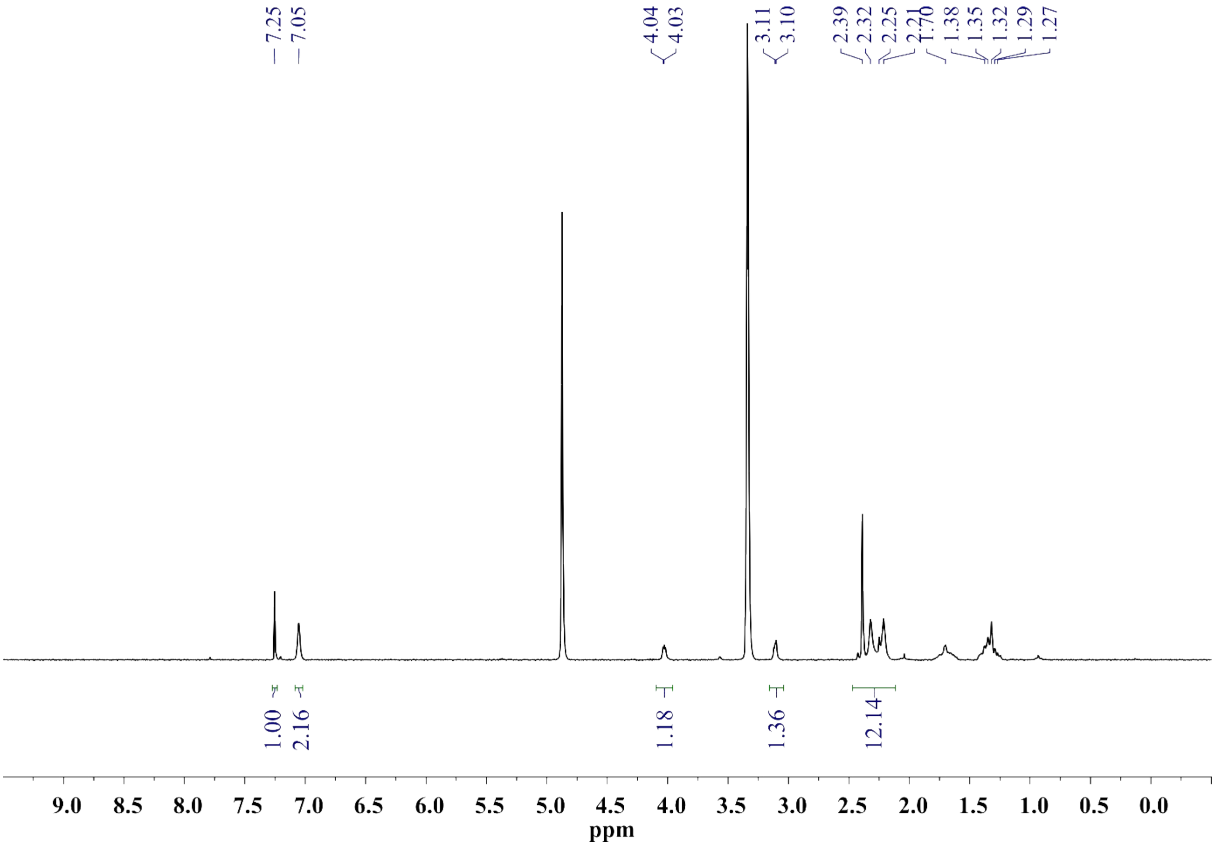

Fig. S2 ^{1}H NMR spectrum of **Ir-IMes** in MeOD-d_4.

NMR spectrometer.

All NMR experiments were performed on a Bruker AVANCE II 300 MHz spectrometer equipped with a BBI 300 MHz W1 5mm z-gradient probe with a BVT-3000 temperature controller. The spectrometer was controlled via the TOPSPIN 3.2. program.

The investigation of interaction between Ir-IMes and 3-ethynylpyridine.

Sample preparation for the investigation of interaction between **Ir-IMes** and 3-ethynylpyridine: The **Ir-IMes** catalysts (cat.) [IrCl(COD)(IMes)] (0.001 mmol, 10 mol% -with respect to 3-ethynylpyridine) and 0.01 mmol 3-ethynylpyridine were dissolved in 1.5 ml MeOD-d_4. Subsequently, the solution ($c_{3\text{-}ethynylpyridine}$=6.7 mM; $c_{cat.}$=0.67 mM) was injected into a Young-type NMR tube. No hydrogen was administered to these samples.

The ^{1}H NMR monitoring: The appropriate sample was inserted into the spectrometer. ^{1}H NMR spectra were recorded (the standard zg Bruker pulse program was employed) from time to time. A multizg macro was used in order to monitor the sample for several hours. At the beginning of the monitoring, for the first hour, ^{1}H NMR spectra were recorded every 10 minutes. Afterwards, for the next 3 hours, the ^{1}H NMR spectra were recorded every 30 min. Finally, for the following 12 hours ^{1}H NMR, spectra were recorded every 1 hour.

Ir-IMes with 3-ethynylpyridine.

For the prepared sample, the creation of the complex **Ir-IMes**: 3-ethynpyridyne was observed on a ^{1}H NMR spectra recorder directly after the mixing of **Ir-IMes** and 3-ethynpyridyne. The analysis of ^{1}H NMR spectra revealed also that the **Ir-IMes** pre-catalyst did not react completely with the excess of 3-ethynpyridyne. It turned out that only ca. 80% of the catalyst was involved in the creation of the [Ir(COD)(IMes)(3-ethynpyridyne)]Cl complex. The complex [Ir(COD)(IMes)(3-ethynpyridyne)]Cl complex was a kinetic product, as the equilibrium was reached directly after the mixing of **Ir-IMes** and 3-ethynylpyridine.

A possible explanation is that the catalyst is not fully consumed and involved in the formation of the ternary complex can result from an acetylene substituent, which modify the electronic properties of the nitrogen atom in the pyridine unit. Please note that the ligands with different substituents may have different binding affinities towards the Ir center in the **Ir-IMes** pre-catalyst.

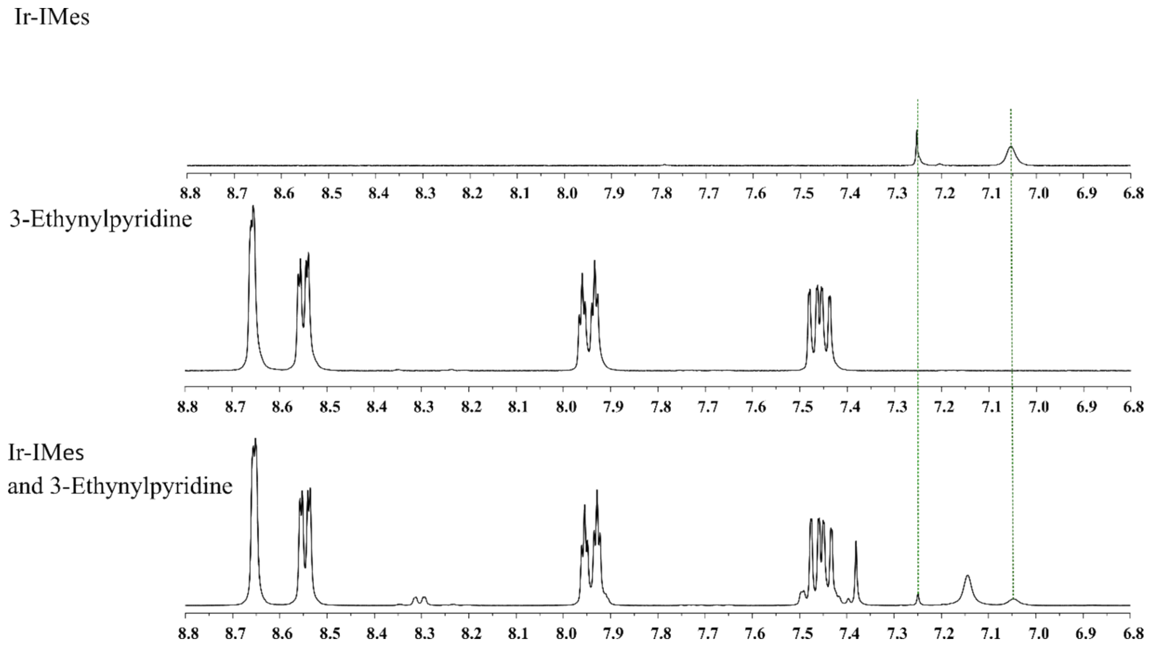

Fig. S3 The superposition of ^{1}H NMR spectra (recorded directly after the preparation of the sample) of pure 3-etynylpyridyne, pure **Ir-IMes** and the mixture of 3-etynylpyridyne with the **Ir-IMes** pre-catalyst.

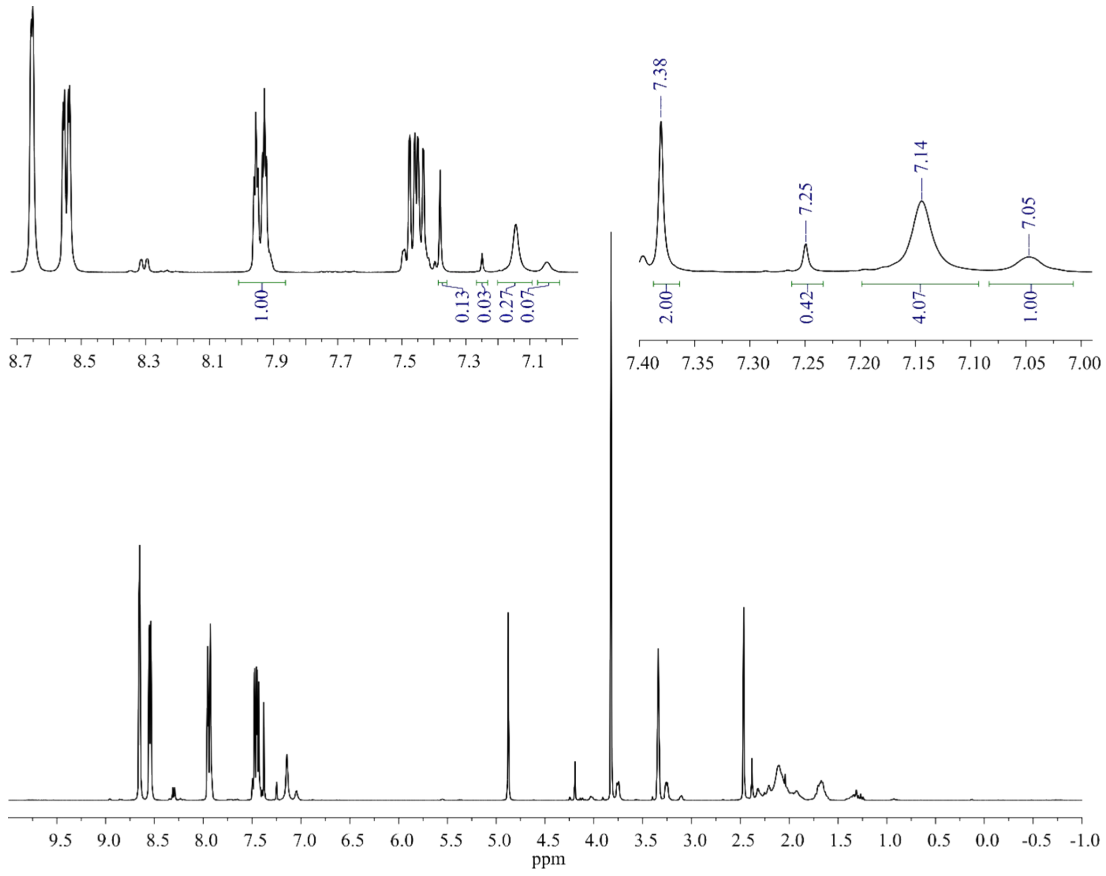

Fig. S4 The ^{1}H NMR spectra (recorded ~12h after the preparation of the sample) of the mixture of 3-etynylpyridyne with the **Ir-IMes** pre-catalyst.

Parahydrogen enrichment.

Preparation of the p-H_2 enriched gas: Hydrogen gas was enriched in its *para* spin isomer at liquid nitrogen temperature (theoretical enrichment yields 50 % *para* isomer) over activated charcoal in a home-built apparatus which was described previously [3]. Please note that the hydrogen gas was allowed to equilibrate over the catalyst at the above-mentioned temperature for ca. three hours before our experiments were started. For the sake of convenience, the hydrogen gas mixture containing 50% of parahydrogen molecules will be called p-H_2.

Hyperpolarization.

Sample preparation for NMR hyperpolarization experiments without DMSO as co-ligand: The appropriate amount of iridium pre-catalyst (**Ir-IMes**) [IrCl(COD)(NHC)] (0.001 mmol, 10 mol% - with respect to 3-ethynylpyridine) and (0.01 mmol) 3-ethynylpyridine were dissolved in 1.5 ml MeOD-d_4. Subsequently, the solution ($c_{3\text{-}ethynylpyridine}$ = 6.7 mM; $c_{\mathrm{cat.}}$ = 0.67 mM); was injected into an NMR tube equipped with a J. Young valve. N_2 purging was carried out prior to the hyperpolarization experiments**.**

Sample preparation for NMR hyperpolarization experiments with DMSO as a co-ligand: The iridium catalysts (**Ir-IMes**) [IrCl(COD)(NHC)] (0.001 mmol, 10 mol% with respect to 3-ethynylpyridine) and 0.01 mmol DMSO were dissolved in 1.5 ml MeOD-d_4. Subsequently, the solution (c_{DMSO}=6.7 mM; $c_{cat.}$=0.67 mM); was injected into an NMR tube equipped with a J. Young valve. p-H_2 purging was carried out several times. Afterwards, the sample was stored under the p-H_2 atmosphere for ca. 30 min. Next, 1 mg (0.01 mmol) of 3-ethynylpyridine was added to the sample. Finally, p-H_2 gas was administered and the hyperpolarization experiments were carried out.

The hyperpolarization experiments. All experiments - including experiments with DMSO, were carried out in the same manner. Each hyperpolarization experiment involves several consecutive cycles.

The definition of cycle. (see Fig. S5): The p-H_2 is administered to the sample under the pressure of 2 bar. Afterwards, the sample is vigorously shaken at the earth's magnetic field (in our case, the shaking was carried out ca. 3 m away from the ultra-shielded NMR spectrometer). Then the sample is inserted into the NMR spectrometer, and ^1H NMR spectrum is acquired with a 90° pulse via the standard zg Bruker pulse program (this type of NMR spectra will be referred to as

hyperpolarized ^{1}H NMR spectra). After 60 s, the next ^{1}H NMR spectrum was recorded (with the same acquisition parameters as the hyperpolarized ^{1}H NMR spectrum). It will be called a relaxed spectrum. Finally, the sample was ejected from the magnet, and the next cycle was conducted in entirely the same way without any delay.

Please not that:

The shaking procedure were carried out 3 m away from the spectrometer. Our previous measurements showed that the contribution of the magnetic field of the spectrometer magnet to the total magnetic field at this position is negligible. One can safely assume that all the experiments were done in the Earth's magnetic field. This has ensured better reproducibility of our results compared to the experiments in the stray field of the magnet that are more sensitive to the changes in the shaking procedure (i.e., in the context of constantly keeping the same position/location of the sample).

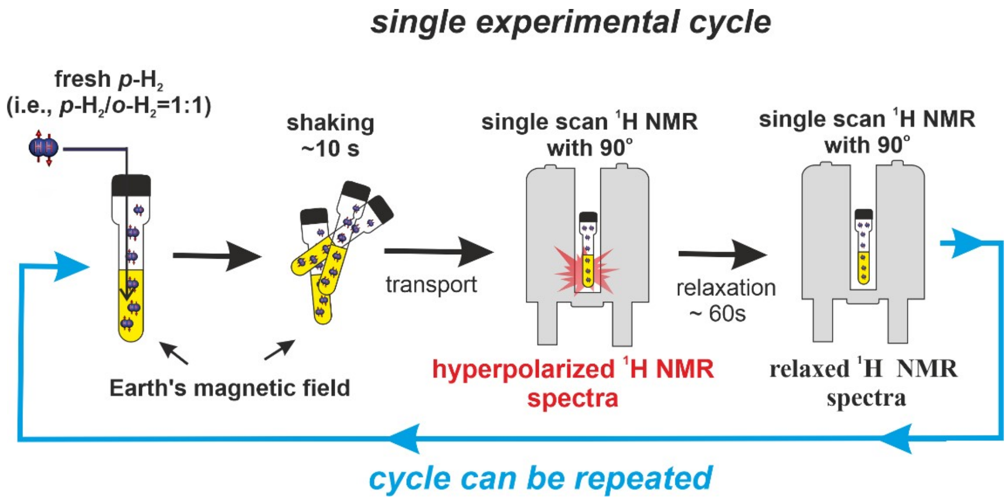

Fig. S5 The basic scenario of the hyperpolarization experiments.

The calculation of the enhancement factors.

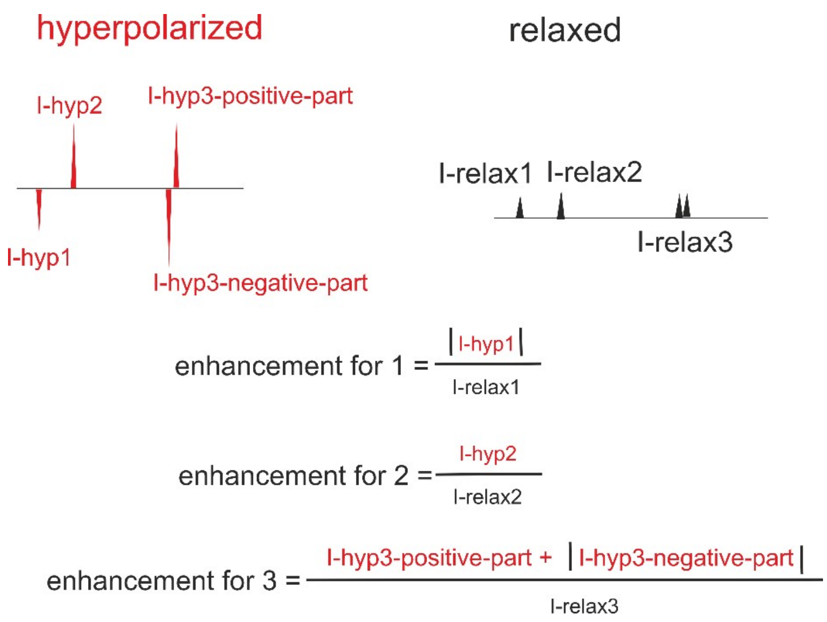

Fig. S6 The method of calculation of the enhancement factors.[2]

Regarding the enhancement factor, one has to note that, in principle, during the one shaking procedure of the NMR sample with p-H_2, three types of hyperpolarized molecules can be generated. In type-a, only the aromatic ring is hyperpolarized; in type-b only the vinyl protons are hyperpolarized; in type-c, both aromatic and vinyl protons are hyperpolarized. The ratio type a:type b: type c is not known. Nevertheless, it has to be noted that the total enhancement factor for SABRE can take into account type a and type b, while the total enhancement factor for hPHIP can take into account type b and type c.

It has to be mentioned that, the enhancement factors determined in successive cycles can vary considerably. This is partly due to the fact that these coefficients are generally small, and even small changes in hyperpolarization parameters - shaking time, transport to the spectrometer - can significantly affect the value of the coefficients.

Hyperpolarization without DMSO as a co-ligand.

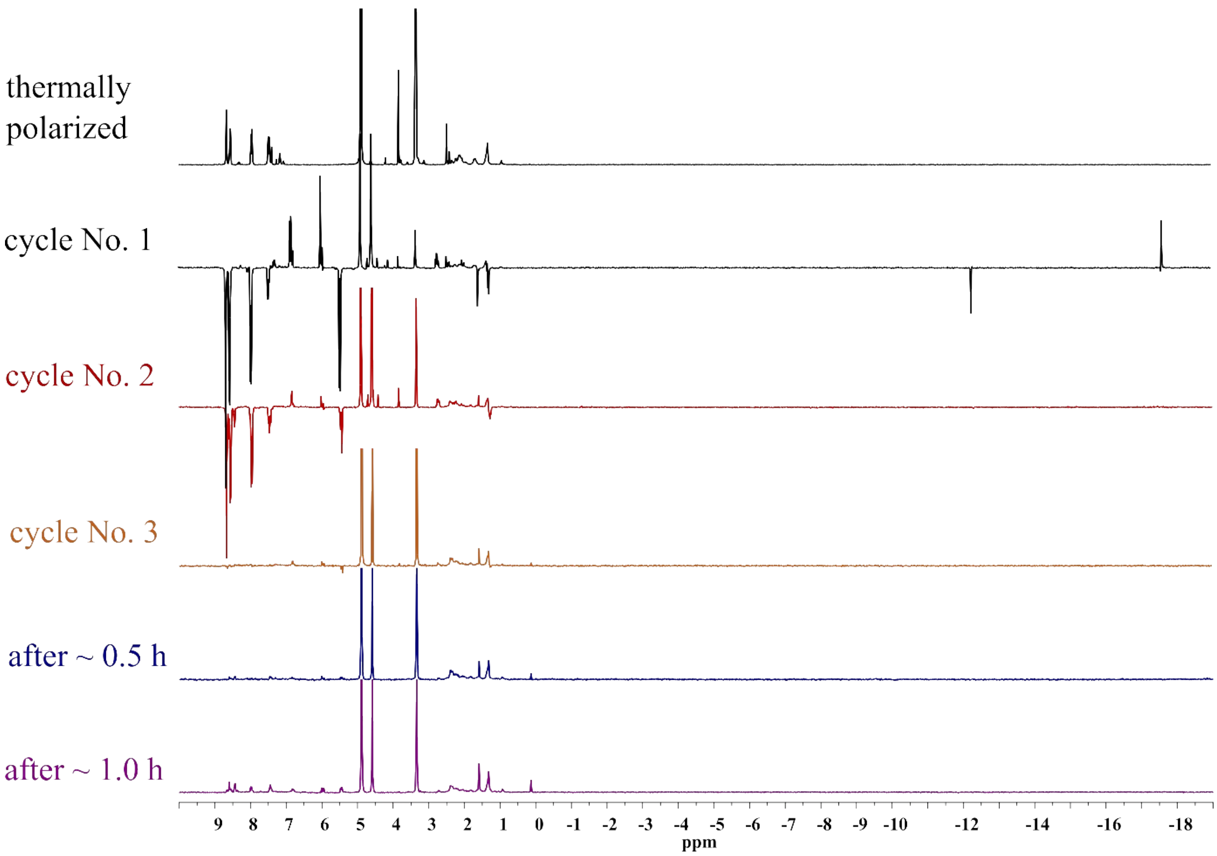

Fig. S7 The superposition of ^{1}H NMR spectra of the mixture of 3-ethynylpyridyne with 10 mol% **Ir-IMes** recorded directly after the administration of p-H_2 in MeOD-d_4 (hyperpolarized spectra) -no hydride signals were observed below -18 ppm.

Table S1 Enhancement factors calculated for aromatic protons (SABRE) and vinyl protons (hPHIP) of 3-ethynylpyridine in the mixture of 3-ethynylpyridyne with 10 mol% **Ir-IMes**.

Time	SABRE enhancement factors				PHIP enhancement factors		
	H1	H4	H2	H3	H_A	H_B	H_C
cycle no. 1	5.19	4.97	4.11	1.11	9.71	21.12	27.83
cycle no. 2	3.68	3.49	2.90	1.03	7.52	2.40	10.68
cycle no. 3	n.d.	n.d.	n.d.	n.d.	n.d.	n.d.	n.d.
after ~ 0.5h	1.00	1.18	0.75	0.93	1.14	0.78	1.28
after ~ 1.0h	1.25	1.11	1.04	1.00	0.95	1.03	0.99

n.d. – not determined.

By comparing the spectrum before and after shaking (see Fig. S8) the tube, it was possible to estimate the amount of substrate that had been hydrogenated. Approximately, 5-10 % of the substrate was consumed during the one cycle. Based on this estimation (the sample contains 0.01mmlo of substrate: 3-ethynylpyridyne), the amount of H_2 consumed in hydrogenation was evaluated to be ~0.1 cm^3 (at 2 bar), while the volume of the NMR tube was approximately ~3 cm^3. Thus, the consumption of H_2 gas during the shaking experiment is negligible in the context of the reduction of pressure, and thus it probably affects SABRE efficiency in a minimal way.

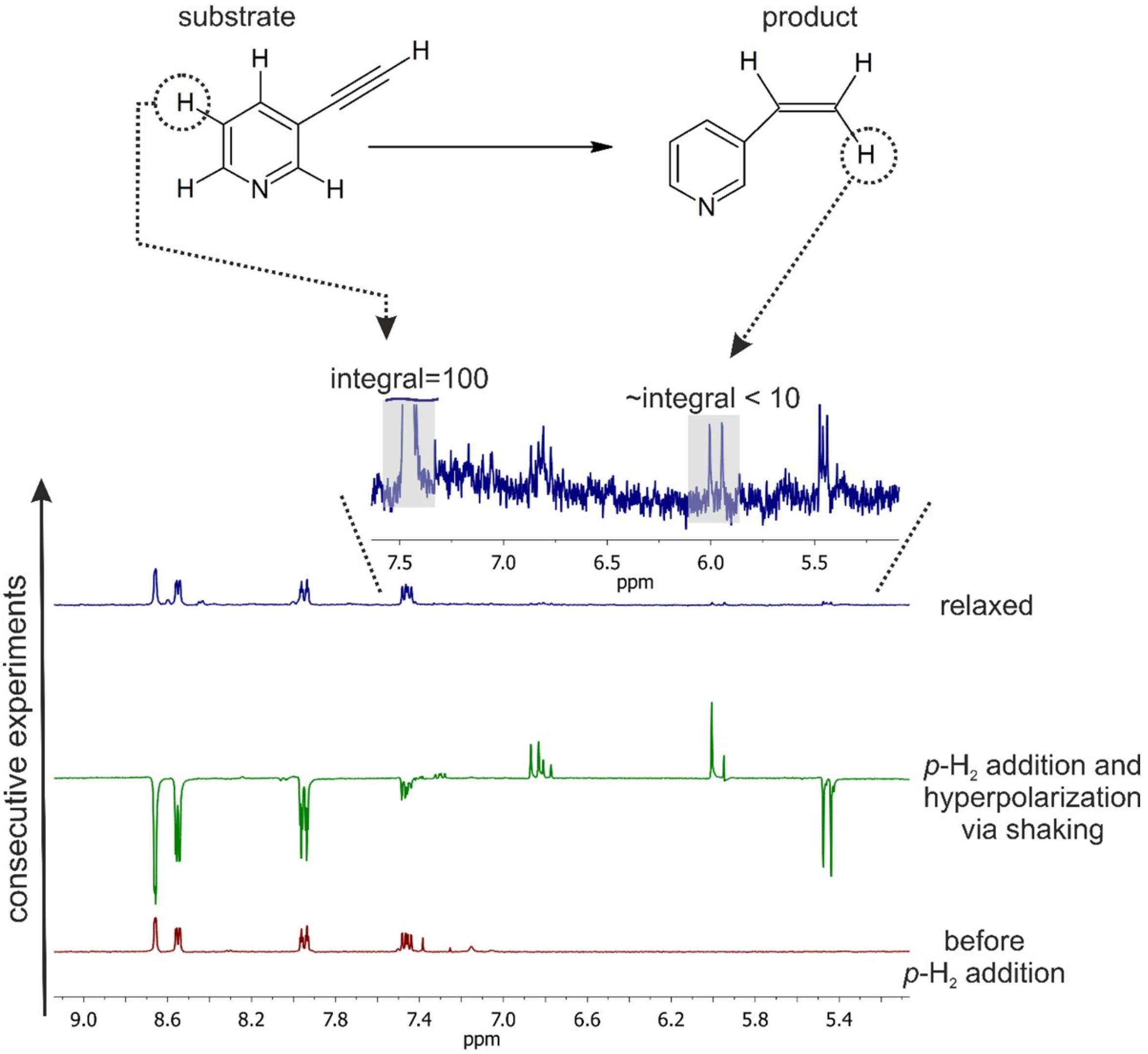

Fig. S8 The estimation of the amount of 3-ethynylpyridyne which is consumed during the one shaking experiment, i.e., one cycle.

The deactivation of SABRE ternary complexes via dimerization and polymerization.

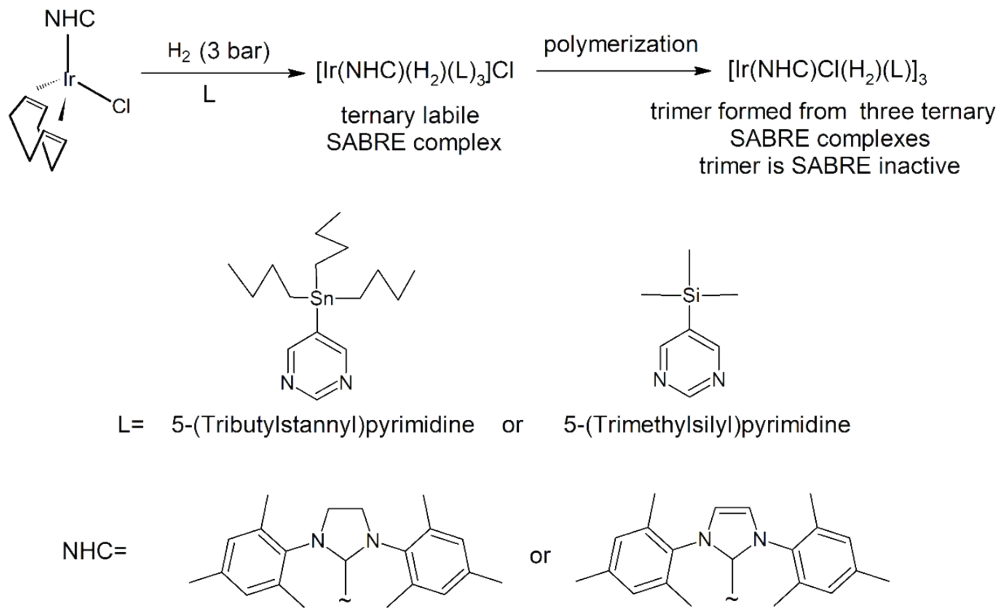

Fig. S9 The deactivation of SABRE ternary complexes with pyrimidine derivatives via polymerization.[3]

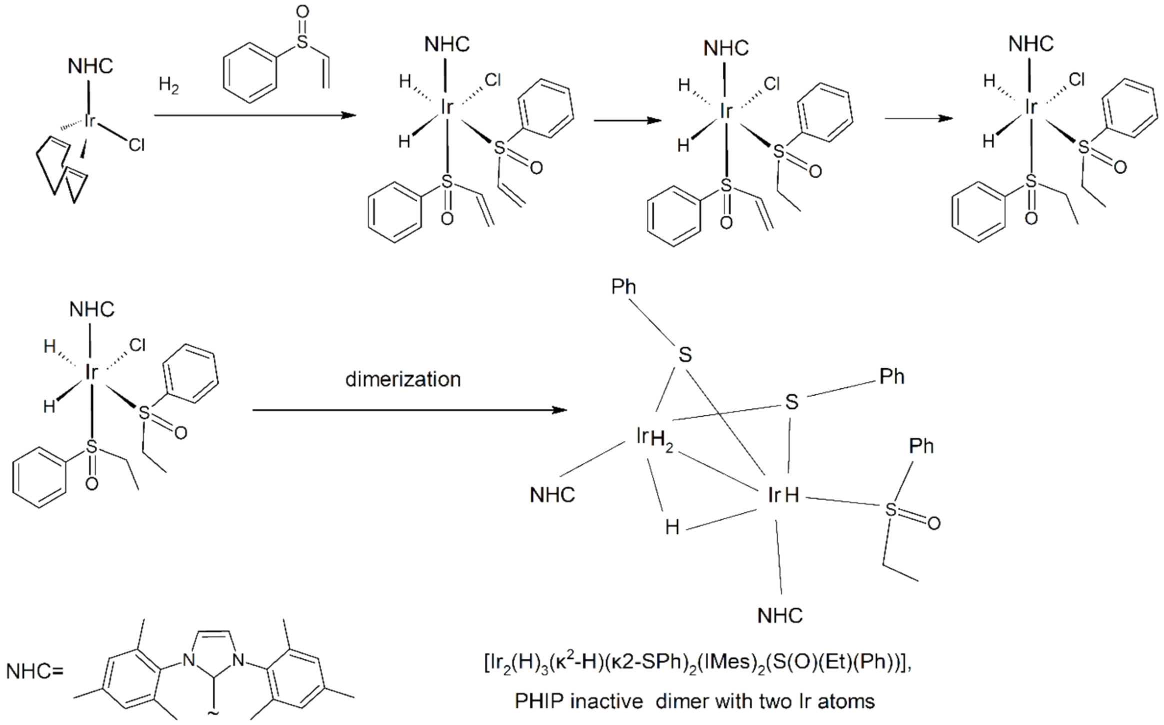

Fig. S10 The deactivation of the hPHIP effect via dimerization.[4]

Hyperpolarization with DMSO as a co-ligand.

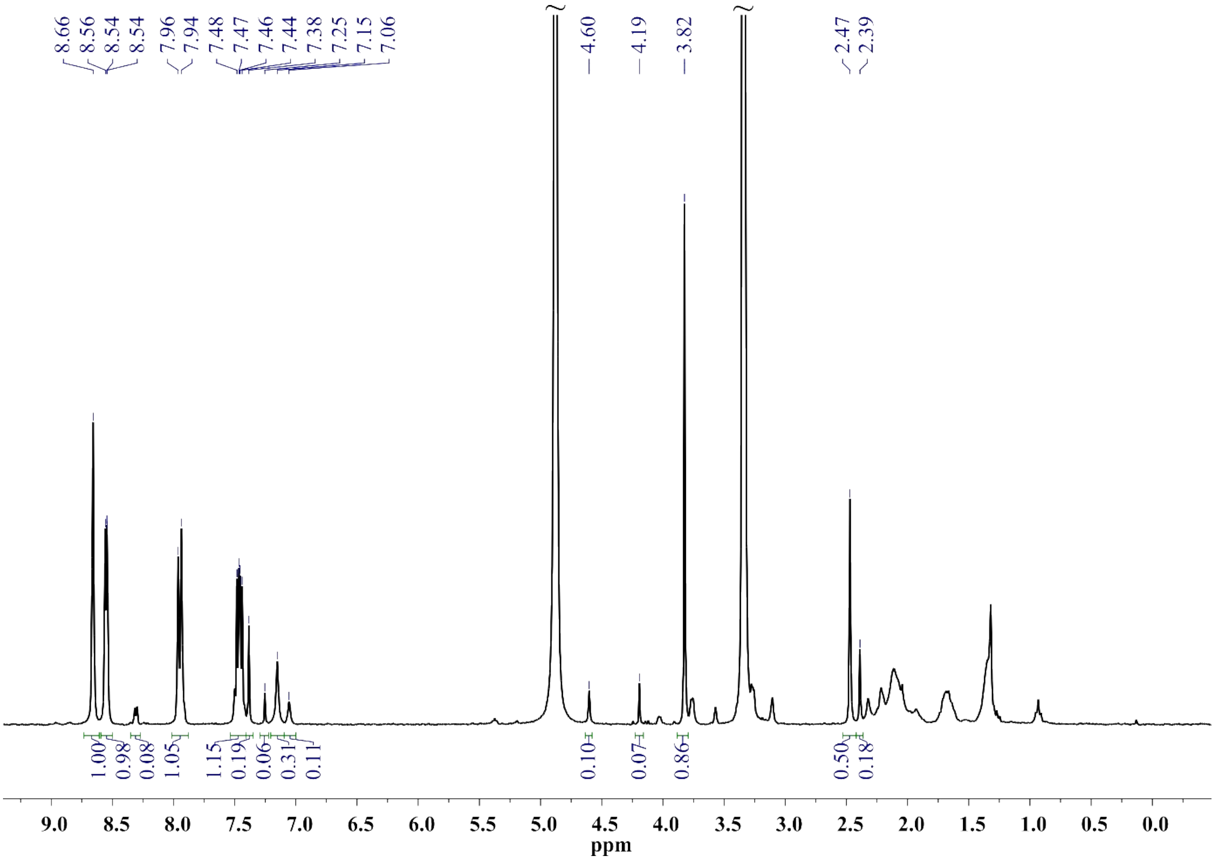

Fig. S11 The ^{1}H NMR spectrum of the mixture of 3-ethynylpyridyne with 10 mol% the **Ir-IMes** pre-catalyst and DMSO as a co-ligand in MeOD-d$_4$.

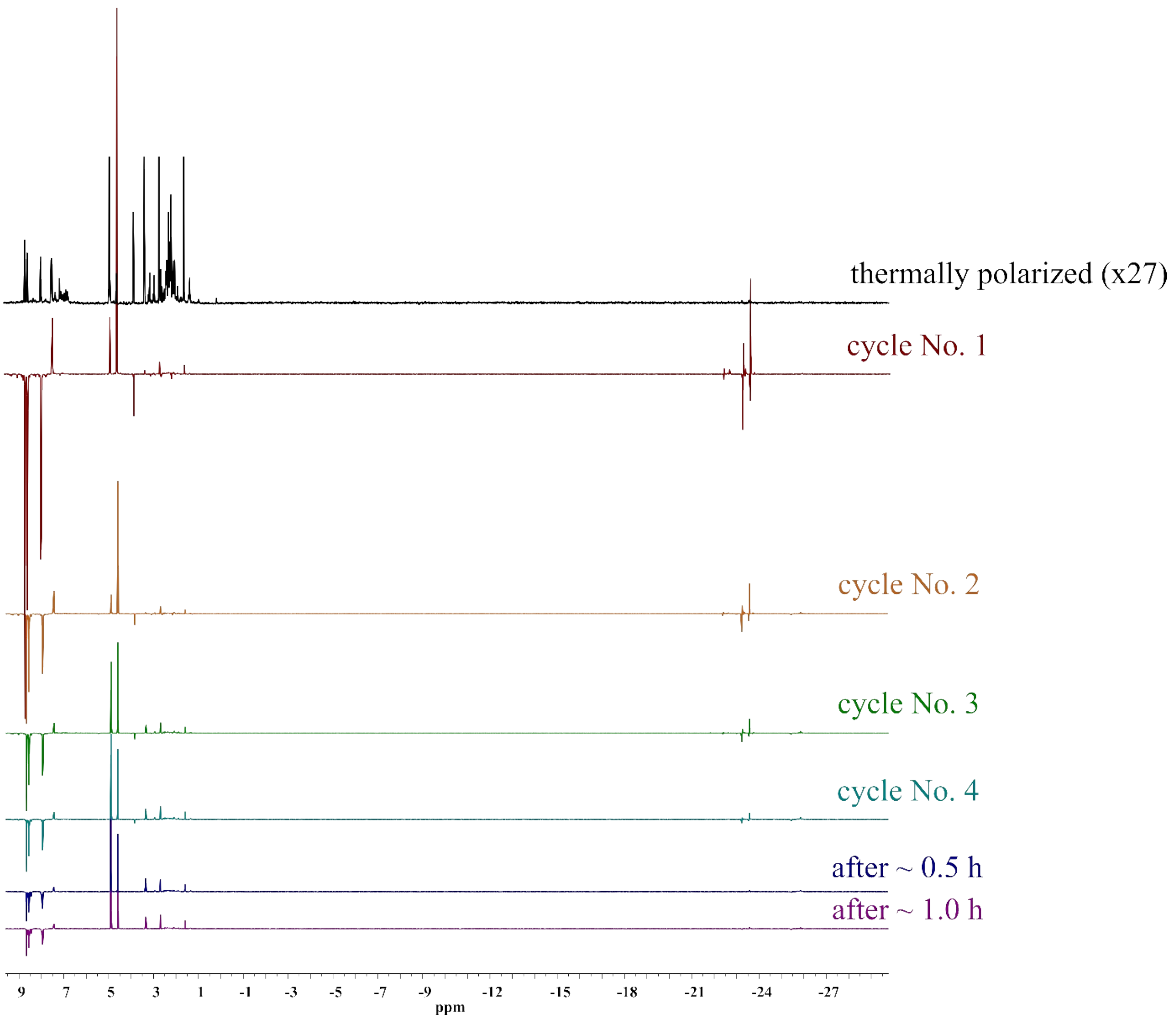

Fig. S12 The superposition of ^{1}H NMR spectra of the mixture of 3-ethynylpyridyne with 10 mol% **Ir-IMes** and DMSO as a co-ligand in MeOD-d$_4$, recorded directly after the administration of p-H_2 (hyperpolarized spectra).

Table S2 Enhancement factors calculated for aromatic protons (SABRE) of 3-ethynylpyridine in the mixture of 3-ethynylpyridyne with 10 mol% **Ir-IMes** and 10 mol% DMSO.

Time	SABRE enhancement factors			
	H1	H4	H2	H3
cycle no. 1	29.7	28.0	21.8	5.39
cycle no. 2	21.7	18.4	14.4	2.68
cycle no. 3	7.88	7.86	5.50	0.97
cycle no. 4	6.39	6.61	4.43	0.58
after ~ 0.5h	4.16	3.99	2.70	0.51
after ~ 1.0h	3.97	3.82	2.56	0.56

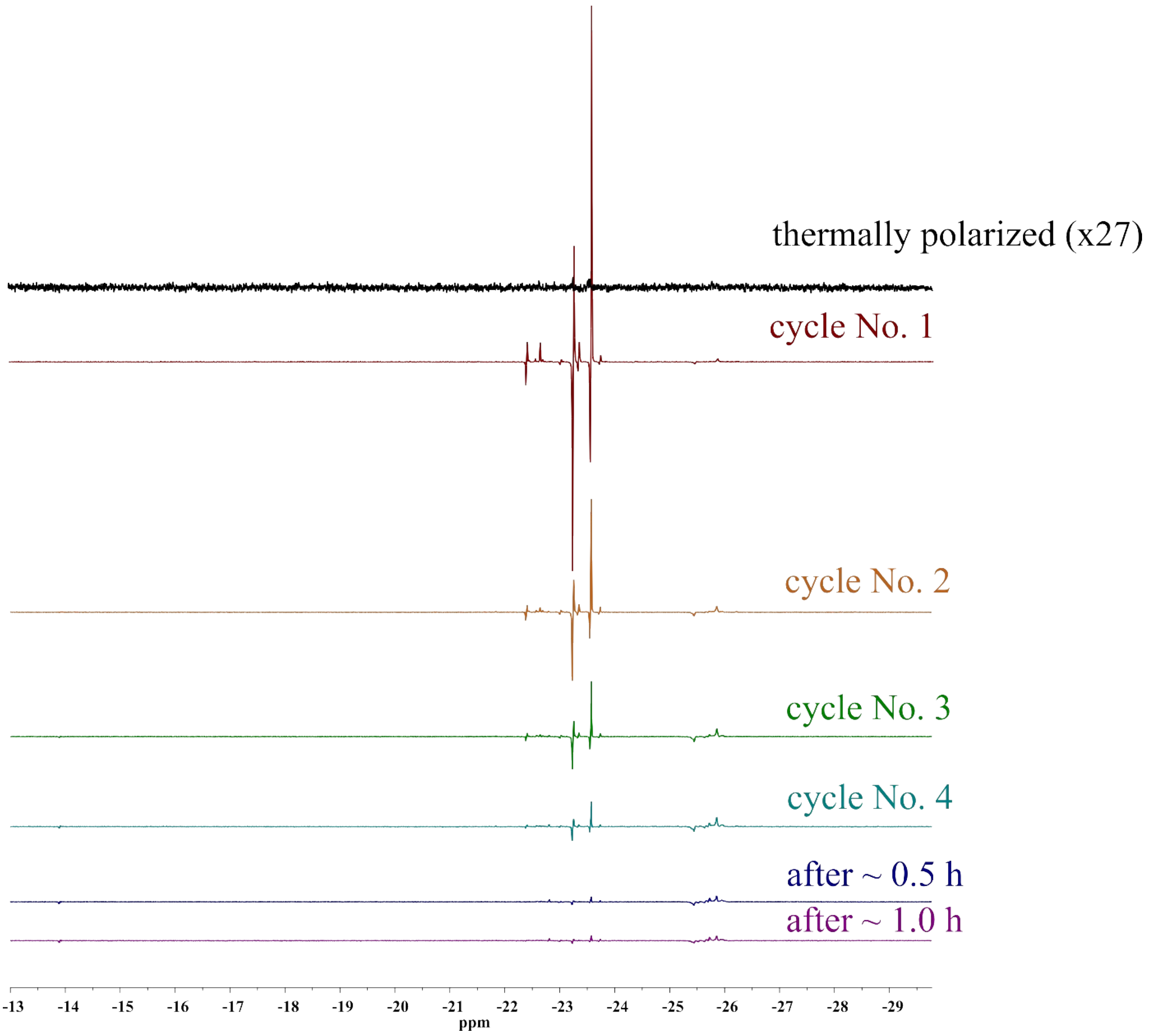

Fig. S13 The superposition of ^{1}H NMR spectra of hydride region of the mixture of 3-ethynylpyridyne with 10 mol% **Ir-IMes** and DMSO as a co-ligand in MeOD-d_4 recorded directly after the administration of p-H_2 (hyperpolarized spectra).

The decay of SABRE activity.

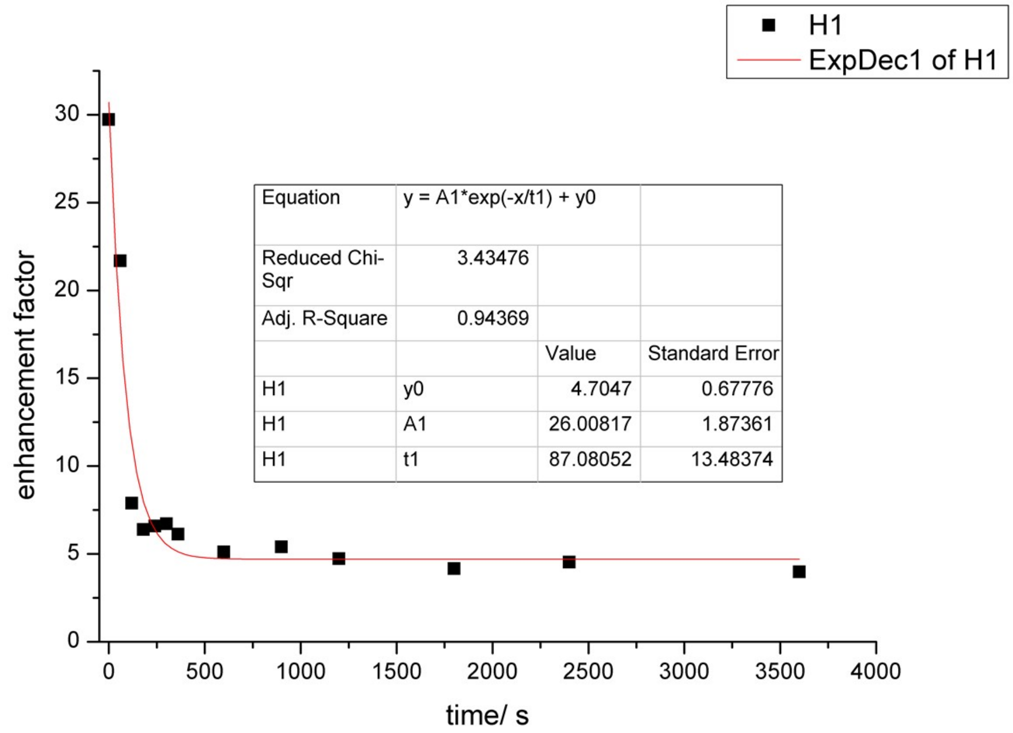

Fig. S14 The decay of SABRE activity: enhancement factors for H1 for SABRE experiments at different time (i.e., different cycles).

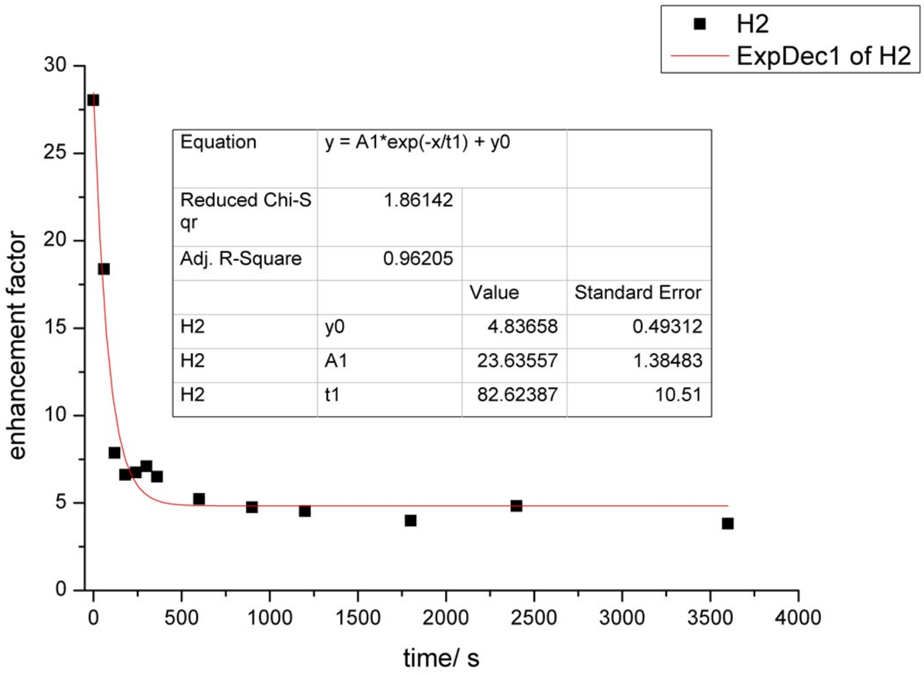

Fig. S15 The decay of SABRE activity: enhancement factors for H2 for SABRE experiments at different time (i.e., different cycles).

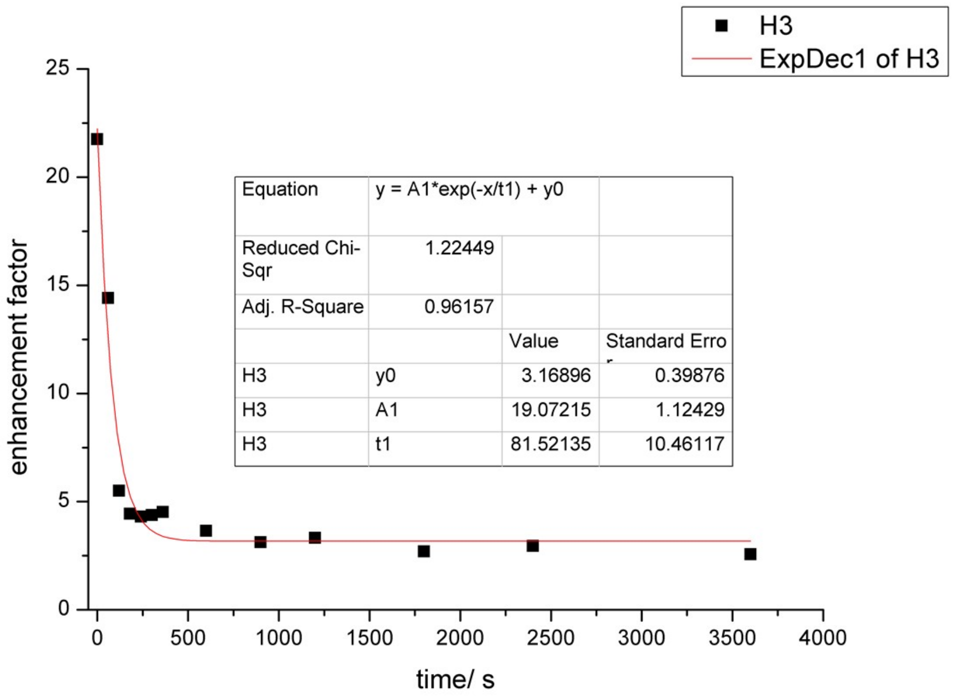

Fig. S16 The decay of SABRE activity: enhancement factors for H3 for SABRE experiments at different time (i.e., different cycles).

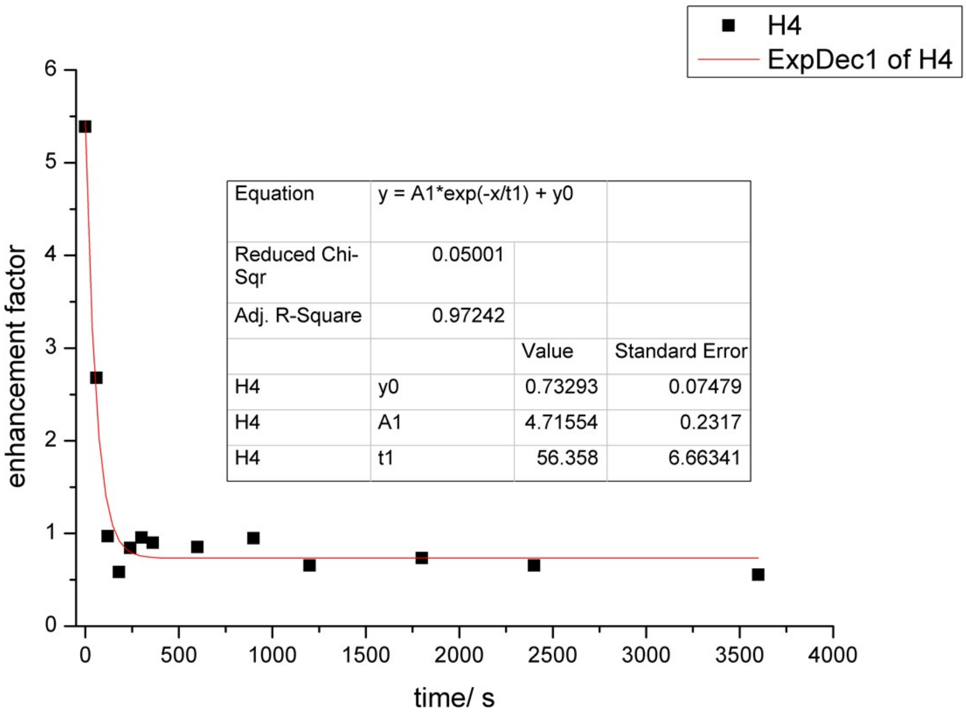

Fig. S17 The decay of SABRE activity: enhancement factors for H4 for SABRE experiments at different time (i.e., different cycles).

References.

[1] I. Kownacki, M. Kubicki, K. Szubert, B. J., Marciniec, *Organomet. Chem.*, **2008**, 693, 321-328.

[2] T. Gutmann, P. Bernatowicz, G. Buntkowsky, J. Frydel, B. Fedorczyk, T. Ratajczyk, *Chem. Eur. J.*, **2015**, 21, 12616–12619.

[3] A. M. Olaru, A. Burt, P. J. Rayner, S. J. Hart, A. C. Whitwood, G. G. R. Green and S. B. Duckett, *Chem. Commun.*, **2016**, 52, 14482–14485.

[4] B. J. Tickner, R. R. Parker, A. C. Whitwood and S. B. Duckett, *Organometallics*, **2019**, 38, 4377–4382.
